# Robot assisted radical prostatectomy in kidney transplant recipients: surgical, oncological and functional outcomes of two different robotic approaches

**DOI:** 10.1590/S1677-5538.IBJU.2018.0308

**Published:** 2019-04-01

**Authors:** Francesco Alessandro Mistretta, Antonio Galfano, Ettore Di Trapani, Dario Di Trapani, Andrea Russo, Silvia Secco, Matteo Ferro, Gennaro Musi, Aldo Massimo Bocciardi, Ottavio de Cobelli

**Affiliations:** 1 Department of Urology, European Institute of Oncology, Milan, Italy; 2 Department of Urology, Niguarda Hospital, Milan, Italy

**Keywords:** Kidney Transplantation, Prostatic Neoplasms, Prostatectomy, Robotics

## Abstract

**Background:**

To date, few series on robot-assisted radical prostatectomy (RARP) in kidney transplant recipients (KTRs) have been published.

**Purpose:**

To report the experience of two referral centers adopting two different RARP approaches in KTRs. Surgical, oncological and functional results were primary outcomes evaluated in the study.

**Material and methods:**

We retrospectively analyzed data from 9 KTRs who underwent transperitoneal RARP or Retzius-sparing RARP for PCa from October 2012 to April 2016. Data were reported as median and interquartile range (IQR). Pre- and postoperative outcomes were compared by non-parametric Wilcoxon signed-rank test. Significant differences were accepted when p ≤ 0.05. Overall survival was assessed using Kaplan-Meier method.

**Results:**

Four KTRs underwent a T-RARP and 5 a RS-RARP. Patient median age was 60 (56-63) years. Charlson comorbidity index was 6 (5-6). Preoperative median PSA was 5.6 (5-15) ng / mL. Preoperative Gleason score (GS) was 6 in 5 patients, 7 (3 + 4) in 3, and 8 (4 + 4) in one. Pre- and postoperative creatinine were 1.17 (1.1; 1.4) and 1.3 (1.07; 1.57) mg / dL (p = 0.237), while eGFR was 66 (60-82) and 62 (54-81) mL / min / 1.73m2 (p = 0.553), respectively. One (11.1%) Clavien-Dindo grade II complication occurred. Two extended template lymphadenectomies were performed, both with nodal invasion. These two patients experienced a biochemical recurrence and were subjected to RT. Two patients (22.2%) had PSMs. Median follow-up was 42 months. Seven patients (77.8%) were continent, 5 (55.6%) were potent. Two (22.2%) patients died during follow-up for oncologic unrelated causes.

**Conclusions:**

Our series suggests that both RARP approaches are safe and feasible techniques in KTRs for PCa.

## INTRODUCTION

In the last decades, a longer and qualitatively better life has been granted to kidney transplant recipients (KTRs) (1).

However, a higher rate of carcinogenesis has been described with an increase of the risk of malignant transformation by three to five folds compared to age-matched controls (2).

In these patients, prostate cancer (PCa) represents the most common tumor among genitourinary malignancies (3). Despite several series have been published, the real incidence of localized PCa in these cohorts remains unclear, ranging from 0.72% to 3.1% (4, 5). Recently, a large population of 123.280 transplant recipients has been investigated and PCa was identified as the most frequent organ-confined neoplasia, diagnosed in 0.82% patients (6).

Although different options have been proposed for the treatment of PCa in KTRs, such as surgery, radiotherapy and brachytherapy (7), radical prostatectomy remains the preferred option. Reasons in favor of surgery could be ascribed to possible complications associated to radiation treatment (RT) in this group of patients (ie. nephritis, ureteral anastomosis strictures, avascular necrosis of the femoral head).

To date, few data have been reported regarding active surveillance (AS) or watchful waiting (WW) for PCa in KTRs (8). However, non-active treatments would be perhaps adopted in the future even in this specific low risk PCa population.

Though in small series, almost all approaches for radical prostatectomy have been described, including retropubic, transperineal, and laparoscopic (4, 9-11). However, radical prostatectomy is more complex in KTRs due to previous peritoneal dialysis, transplant surgery, graft location and immunosuppression. In 2008, Jhaveri et al. described the first robot assisted radical prostatectomy (RARP) in a patient with a kidney graft. To our knowledge, only limited series have been published about RARP in KTRs (4, 5, 7, 11-15).

The aim of the current study is to evaluate safety, feasibility and efficiency of two different RARP techniques in KTRs, performed in two high-volume referral centers, and to describe intra- and post-operative outcomes, analyzing short- and medium-term follow-up oncological and functional outcomes.

## MATERIALS AND METHODS

From October 2012 to May 2016, nine patients previously subjected to renal transplantation underwent RARP. Four of them, experienced a trans-peritoneal RARP (T-RARP); five a Retzius-sparing RARP (RS-RARP); all of them were diagnosed with a localized PCa.

All data were prospectively collected in two different customized databases and retrospectively analyzed.

Baseline demographic features, surgical, oncological and functional outcomes were investigated and complications were evaluated according to the Clavien-Dindo scale (16).

Potency was defined as erections allowing satisfying penetrations. Continence recovery was assessed according to ICIQ criteria (17).

### Description of the techniques

Patients were placed in lithotomy position, with a 27 to 30 degrees Trendelemburg inclination. All pressure points were carefully padded in order to avoid vascular and nervous injuries. Before the port placement, a bladder catheter was positioned.

A standardized four-arm robotic configuration was used in all patients, either with the robotic da Vinci® Si or Xi systems, both placed caudally between the legs: a total of 6 ports were used, 3 for the robotic arms, 1 for the camera and 2 for the bedside assistant (one of 12mm and one of 5mm).

**Figure 1:**
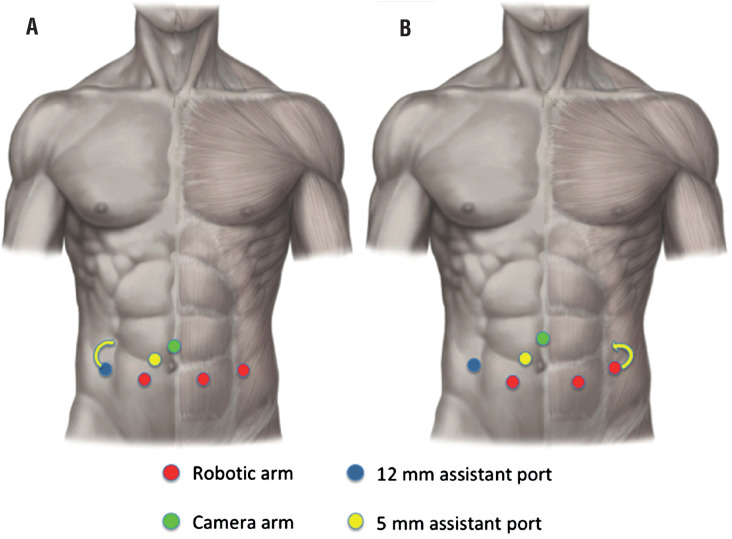
Robotic and assistant port positioning for T-RARP. A) The placement of the 12 mm assistant port was modified, medially and cranially respect to the standard set, in case of right kidney graft location. B) The placement of the third robotic arm was modified, medially and cranially respect to the standard set, in case of right kidney graft location.

**Figure 2:**
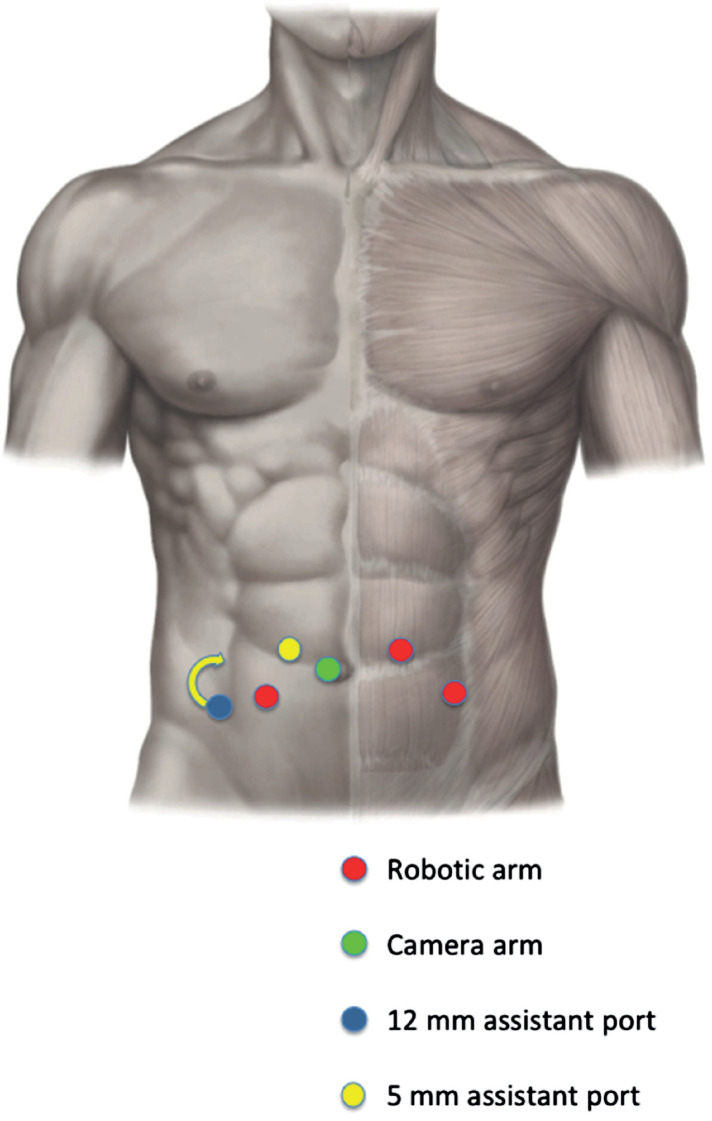
Robotic and assistant port positioning for RS-RARP. The placement of the 12 mm assistant port was modified, medially and cranially respect to the standard set, in case of right kidney graft location.

The placement of the third robotic arm or the 12mm assistant port was modified, medially and cranially respect to the standard set, in the renal transplant recipients to avoid trauma to the graft as represented in Figures 1 and 2.

Lymphadenectomies were carried out according to the preoperative risk of lymph node invasion assessed according to the updated Briganti nomogram (18).

### European Institute of Oncology

In two KTRs, the bedside assistant stood on the left side, instead of on the right, to avoid trauma to the right positioned kidney graft during the laparoscopic instruments insertion.

The peritoneal incision, external to the umbilical ligaments, was tailored according to the transplant side; the remaining procedure was conducted equally to our standardized RARP procedure.

All patients underwent a nerve sparing procedure, two patients only anterograde and two anterograde and retrograde.

In all patients, a posterior musculo-fascial reconstruction after radical prostatectomy was performed as described by Coelho et al. (19) and no lymphadenectomies were carried out.

### Niguarda Hospital

The bedside assistant was placed on the right side of the patient. In case of a right-sided kidney transplant, the 12mm assistant port was placed more cranially. After the docking, the Retzius-sparing surgery was conducted as previously described (20), without modifications from the standard protocol: the peritoneal incision was performed on the rectovesical pouch, where the surgical field was intact from previous surgeries and the kidney was placed distant. No posterior reconstruction techniques have been used.

One monolateral and one bilateral lymphadenectomy were performed, adopting an extended template. The main limitation in performing a bilateral template was ascribed to the previous vessels dissection and vascular anastomosis for the kidney graft. Moreover, our dissection resulted very difficult due to the healing adhesions found in the perinodal fat.

### Statistical analysis

Data are reported as median and interquartile range (IQR). Pre- and postoperative outcomes were compared by non-parametric Wilcoxon signed-rank test. Significant differences were accepted when p ≤ 0.05. Overall survival (OS) was assessed using Kaplan-Meier method.

Statistical analysis was performed using SPSS, version 20 (IBM, New York, NY, USA).

## RESULTS

Pre-, intra- and postoperative outcomes are showed in Tables 1 and 2. Data are reported as median and interquartile range (IQR). Patient age at surgery was 60 (56-63) years, with a BMI of 25.7 (24.2-27.7) kg / m^2^. The interval between renal transplantation and RARP was 9 years (6-22): renal graft was sited on the right iliac fossa in 7 (77.8%) patients and on the left for the remaining 2 (22.2%). Charlson comorbidity index was 6 (5-6). In the preoperative assessment median PSA resulted of 5.6 (5-15) ng / mL, while the clinical stage was T1c in 4 (44.5%) patients, T2a in 3 (33.3%), T2c and T3b for the remaining two. At the prostate biopsy, the Gleason score was established as 6 (3 + 3) in 5 (55.6%) patients, 7 (3 + 4) in 3 (33.3%) patients, and 8 (4 + 4) in the remaining one (11.1%). Among the 5 patients diagnosed with a GS of 6 (3 + 3), 2 had oncologic characteristic adequate for AS according to PRIAS criteria (21), but both refused the surveillance program. Due to preoperative serum creatinine of 6.7 mg / dL, one (11.1%) patient was subjected to hemodialysis the day before radical prostatectomy. No significant difference was described comparing serum creatinine levels before and after surgery, accounted respectively for 1.17 (1.1-1.4) and 1.3 (1.07-1.57) mg / dL (p = 0.237). Furthermore, no significant difference was described for eGFR calculated as 66 (60; 82) and 62 (54; 81) mL / min / 1.73m2 respectively before and after surgery (p = 0.553). In two (22.2%) patients chronic kidney disease status (CKDS) worsened of 1 point after surgery, while in one (11.1%) patient we detected a 1-point improvement. No intraoperative complications were reported. Overall, operating time was 160 (145-183) minutes, with an estimated blood loss of 100 (100-200) mL. Six (66.7%) patients were subjected to a nerve sparing procedure. Prostate weight was 40 (39-45) g. Median hemoglobin decrease was 2.6 (2.15-3.2) and no blood transfusion was necessary. One (11.1%) Clavien-Dindo grade II complication was described due to a systemic inflammatory syndrome for urinary tract infection. The hospital stay consisted in 4 (3-6) days, while the days of catheterization were 7 (6-8) days.

**Table 1:** Robotic and assistant port positioning for T-RARP.

Variables		
Age, yrs (median, IQR)	60	(56-63)
BMI, kg/m^2^ (median, IQR)	25.7	(24.2-27.7)
CCI (median, IQR)	6	(5-6)
Time from transplantation, yrs (median, IQR)	9	(6-22)
Creatinine, mg/dL (median, IQR)	1.17	(1.1;1.4)
PSA, ng/mL (median, IQR)	5.6	(5-15)
Gleason score Sum (N°, %)	(3+3)	5 (55.6%)
	(3+4)	3 (33.3%)
	(4+4)	1 (11.1%)
Clinical Stage (N°, %)	T1c	4 (44.5%)
	T2a	3 (33.3%)
	T2c	1 (11.1%)
	T3a	1 (11.1%)

Abbreviations: **yrs =** Years; **IQR=** Interquartile range; **BMI =** Body mass index; **CCI =** Charlson Comorbidity Index; **PSA =** Prostate Specific Antigen

**Table 2:** Robotic and assistant port positioning for RS-RARP.

Variables		
Operative time, min (median, IQR)	160	(145-183)
EBL, mL (median, IQR)	100	(100-200)
Length of stay, days (median, IQR)	4	(3-6)
N° of LADs (N°, %)	2 (22.2%)	
N° of positive LADs (N°, %)	2 (22.2%)	
Days of catheterization (median, IQR)	7	(6-8)
Complications by Clavien scale (N°, %)	Grade II: 1 (11.1%)	
Gleason score Sum (N°, %)	(3+3)	4 (44.5%)
	(3+4)	3 (33.3%)
	(4+3)	2 (22.2%)
Pathologic Stage (N°, %)	pT2a	1 (11.1%)
	pT2c	6 (66.7%)
	pT3a	1 (11.1%)
	pT3b	1 (11.1%)
PSMs (N°, %)	2 (22.2%)	
BCR (N°, %)	2 (22.2%)	
Potency recovery rate (N°, %)	5 (55.6%)	
Continence recovery rate (N°, %)	7 (77.8%)	
Last follow up serum creatinine, mg/dL (median, IQR)	1	(1-2.7)

Abbreviations: **IQR =** Interquartile range; **EBL =** Estimated blood loss; **LADs =** Lymphadenectomies; **PSMs =** Positive surgical margins; **BCR =** Biochemical recurrence

No changes in immunosuppressive regimes were reported in pre- or postoperative periods. Regarding calcineurin inhibitors (tacrolimus or cyclosporine), serum levels were daily obtained during the hospital stay and therapy was adjusted as necessary. Pathological analysis reported Gleason score 6 (3 + 3) in four patients (44.5%), 7 (3 + 4) in three (33.3%) and 7 (4 + 3) in the other two (22.2%). One patient (11.1%) was pT2a, six patients (66.7%) pT2c, one (11.1%) pT3a and the last one (11.1%) pT3b. In two patients an extended lymph node dissection was performed, and in both patients a lymph node invasion was assessed. In one case a bilateral lymphadenectomy was performed with a lymph node yield of 19 nodes, 3 of them positive for neoplastic invasion. In the other case, a monolateral lymphadenectomy was conducted with a lymph node yield of 3 nodes, 1 of them positive for neoplastic invasion. These two patients experienced a biochemical recurrence (BCR) (PSA > 0.2 ng / mL) and were subjected to RT. Two patients (22.2%) had positive surgical margins, one of them was focal. All patients reached at least 12 months of follow-up (median 42 months); 7 (77.7%) were continent, the remaining 2 patients were affected by a moderate incontinence; 2 (22.2%) patients were potent, while other 3 (33.3%) of them reached a potency recovery with PDE-5 inhibitors administration. At last follow up median serum creatinine accounted for 1 (1; 2.7) mg / dL, and no significant difference was described with pre preoperative one (p = 0.237). Two (22.2%) patients died for oncologic unrelated causes.

## DISCUSSION

Although widely debated, a greater incidence of PCa was described in transplanted patients (22), with higher frequencies of advanced malignancies and worse disease specific survival (23).

Moreover, thanks to immunosuppression regimen improvements, survival expectancy after kidney transplantation has increased of more than 10 years, (12) enlarging the number of recipients older than fifty years old, and making PCa handling as important as in general population (1).

Generally, alteration of normal tissue's planes caused by previous retroperitoneal surgery and pelvic location of kidney graft are frightening factors that preclude RT in order to avoid adverse events (ie. ureteral stenosis, actinic pyelonephritis and gastrointestinal toxicity) (5, 24).

In 2004, Mouzin et al. published a study in which 8 KTRs underwent external beam RT as primary therapy for localized PCa. In two patients (25%) a significant obstruction of the terminal ureter was described, and one patient (12.5%) had a decrease in renal graft function (24). Moreover, two patients (25%) had BCR after a mean follow-up period of 28 months.

On the contrary, more recently Iizuka et al. showed no severe adverse events in 4 KTRs treated with RT (5).

Typically, localized PCa in KTRs had been treated performing a radical retropubic-prostatectomy (9). However, a surgical treatment could not be thought free from challenges and complications. Most common hitches were graft injuries, including also ureter and vessels, high incidence of intraperitoneal adhesions in patient subjected to peritoneal dialysis, difficulties in performing a vesical-urethral anastomosis due to bladder descent limitation caused by the shortness of transplant ureter (25).

In 2006, Shah et al. described the first series of laparoscopic radical prostatectomy (LRP) in KTRs (26). Despite some studies depicted LRP as safe and feasible in the treatment of PCa in transplant recipients, Robert et al. reported an incidence of rectal injury clearly higher than in general population (22.2 vs. 1.8%, p = 0.022) (27).

Since its advent, robotic approach overcame the technical limitations that have characterized the laparoscopic surgery. Particularly in presence of pelvic graft, the wristed instruments allow for easier suturing and dissection avoiding the graft hindrance flexing over it.

In 2008, Jhaveri et al. reported the first case of the RARP in KTRs (4). Since then, few studies, with small cohort, were published regarding the use of robotic approach to treat localized PCa in this population (Table-3).

**Table 3:** Overall survival Kaplan-Meier curve analysis.

Authors	Year	N° of patients	Surgical approach	Operating time, min	Estimated blood loss, mL	Complications Clavien-Dindo	Hospitalization, days	Catheterization, days	PSM, n° (%)	BCR, n° (%)
Jhaveri et al.	2008	1	Transperitoneal	200	400	No	2	7	No	No
Smith et al.	2011	3	Transperitoneal	322	75	No	2.3	-	1 (33%)	No
Polcari et al.	2012	7	Transperitoneal	186	-	Grade II: 3 1 Haematuria 1 Urosepsis 1 Atrial fibrillation	1.8	8.1	2 (28.6%)	1 (14.3)
Wagener et al.	2012	1	Transperitoneal	220	300	No	-	(4 weeks)	No	No
Ghazi et al.	2012	1	Transperitoneal	130	125	No	-	10	No	-
Le Clerc et al.	2015	12	Transperitoneal	241	648		-	-	3 (27.3)	2 (16.7)
Moreno et al.	2015	4	Transperitoneal	196	-		3.2	10	2 (50)	1 (25)
Iizuka et al.	2016	3	Transperitoneal	162	52	Grade II: 1	-	9.3	No	1 (33.3)
Mistretta et al.	2017	9	Cumulative:	160	100		4	7		
			T-RARP: 4	170	100	No	5	6	No	No
			RS-RARP: 5	150	100	Grade II: 1 1 Urosepsis	3	8	2 (22.2%)	2 (22.2)

Abbreviations: **RARP =** Robot-assisted radical prostatectomy; **T-RARP =** Transperitoneal-RARP; **RS-RARP =** Retzius Sparing-RARP; **PSMs =** Positive surgical margins; **BCR =** Biochemical recurrence

To our knowledge, our series represents the second largest cohort of KTRs treated with RARP, and the only multi-institutional study that includes two different techniques.

Despite the aforementioned advantages, RARP remains a challenging surgery in these patients. Several authors have described some technical modifications necessary to overcome the graft's impediment; for example, Smith et al. and Polcari et al. adopted assistant ports placement contralateral to renal graft (12, 13), while Ghazi et al. and Moreno et al. modified the 6 ports setting into a 5 ports arrangement, performing RARP without a robotic arm. (7, 14); some others, instead, (11, 15) proved the modification of the port sites to be useless.

In our series, during T-RARP assistant, ports were placed contralateral to renal graft, and, as suggest by Jhaveri et al. (4), the robotic arm on the side of the graft was slightly lifted up in order to avoid injuries.

For the RS-RARP instead, only the robotic arm on the side of the graft was moved cranially and laterally respect to the standard setting.

Despite the graft hindrance and the setting modifications, we described comparable operative times in respect to the standard RARP. Furthermore, no intraoperative complications were reported and no blood transfusions were required.

Previous studies reported variable rate of complications (43%), (4, 5, 7, 11-15) maybe results of the small samples size, including urosepsis, hematuria, atrial fibrillation, conversion to laparotomy.

In the current study, we reported only a single case (11.1%) of urosepsis, promptly resolved with appropriate therapy. Noteworthy, no surgical or medical injuries were assessed regarding graft function. In fact, no significant differences in serum creatinine levels were observed between pre- and postoperative settings (p = 0.237), or at the last follow-up (p = 0.237). Similarly, no significant differences were found regarding pre- and postoperative CKDS status.

Regarding the oncological outcomes, pathological analysis described a Gleason score 6 (3 + 3) in 44.5% of patients, 7 (3 + 4) in 33.3% and 7 (4 + 3) in the 22.2%, describing 4 upgrading and 2 downgrading in respect to Gleason score assessed at preoperative prostatic biopsy.

These data underline the problem of RARP for low risk PCa patients, particularly in frail patients such as KTRs. Few studies are reported regarding AS for PCa in transplant recipients. In particular, AS was described only in one study for one patient with low-risk disease (28), WW in one study for four patients in total (29).

In our series AS was proposed to the two patients that satisfied preoperative PRIAS criteria (21), but both of them refused the surveillance program. Although, the immunomodulation and the subsequent impaired spontaneous cancer control should be considered in this population.

Despite the short follow-up and the small size of the series, our study described oncologic outcomes concordant with the ones reported in literature. In the general population, PSM rate of patient treated with RARP for localized PCa ranged from 14.1% to 29% (30). In the previous studies regarding RARP in KTRs PSM rate was reported as 0-50% (4, 5, 7, 11-15). In our study two patients (22.2%) had a PSM, one of them focal.

In general population, BCR free survival rates at 3 years were reported at 96.3% (31). In our study, two patients (22.2%) experienced BCR and underwent radiotherapy. These data are similar to those of previous series of KTRs subjected to RARP. Moreover, high PSA levels and palpable neoplasia at digital rectal examination had been assessed in these two patients before surgery and a nodal invasion was determined at final histology.

**Figure 3:**
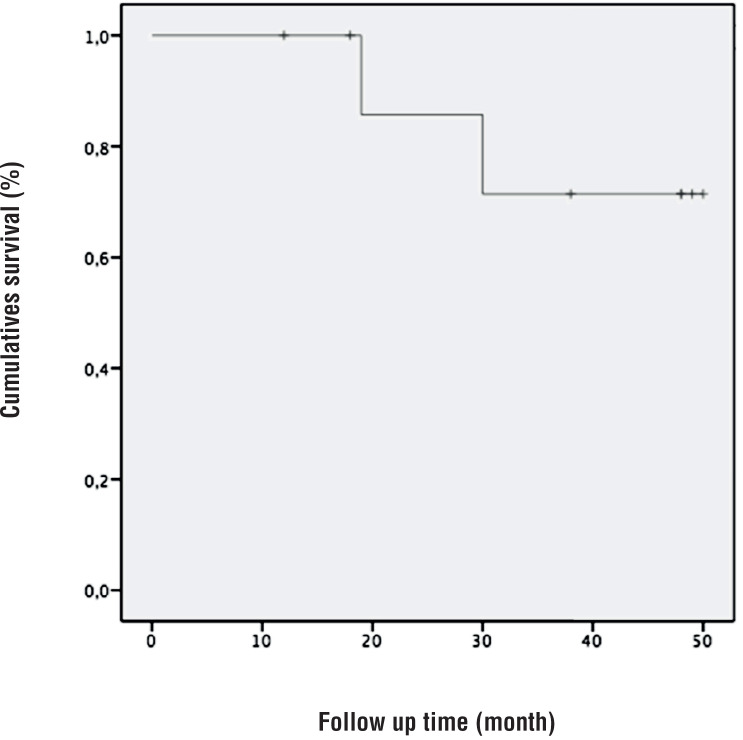
Overall survival Kaplan-Meier curve analysis.

Lastly, two patients (22.2%) died due to cancer unrelated causes. Curve regarding overall survival is showed in Figure-3.

However, similarly to PSM rate and BCR, it is difficult to analyze data regarding survival rate, and these high frequencies could be attributed to the limited number of cases in the series.

A good continence recovery was described in previous studies (4, 7, 11-13). Our data agree with the former assessing a complete continence recovery of 7 out 9 patients (77.8%). In the remaining two patients a moderate incontinence (17) was described. Noteworthy, one of these patients was subjected to adjuvant RT influencing the continence recovery.

Our study is the first reporting data on erectile function recovery. KTRs seem to be associated with higher rate of erectile dysfunction (32). The nerve sparing approach could result even more challenging if performed in KTRs.

In general population, potency recovery rate at one year after the surgical procedure was assessed ranging from 29.6% to 77.6% (33, 34). In the current study 6 patients (66.7%) received a nerve-sparing procedure (4 bilateral, 2 monolateral), and 5 (55.6%) achieved a potency recovery, with or without PDE5i intake, sufficient to have sexual intercourses.

The current study has some limitations due to the small sample size. However, although in future the incidence of PCa could increase in KTRs, nowadays it remains limited. This issue imposes to treat the pathology in referral high volumes centers, by expert surgeons.

Second limitation is the retrospective nature of the analysis. However, as previously stated the rarity of KTRs patients affected by PCa makes difficult to complete a randomized prospective trial.

## CONCLUSIONS

The current study's results suggest that both RARP approaches adopted at our institutions may be safely applied in KTR patients. A low morbidity and overall good surgical outcomes were reported without any major complication. Oncologic and functional outcomes showed are comparable with those of general population patients subjected to RARP. However, robot-assisted radical prostatectomy still remains a challenging surgery in KTRs, and should be performed by expert robotic surgeons, in a tertiary referral center. Furthermore, a more consistent patient cohort is needed to confirm our results.

## ABBREVIATIONS

BCR: = biochemical recurrence
CKDS: = chronic kidney disease status
IQR: = interquartile range
KTRs: = kidney transplant recipients
PCa: = prostate cancer
RARP: = robot-assisted radical prostatectomy
RS-RARP: = Retzius sparing RARP
RT: = radiation treatment
T-RARP: = transperitoneal RARP
